# Supplementary material for: Conditional chemoconnectomics (cCCTomics) as a strategy for efficient and conditional targeting of chemical transmission
Source: eLife. 2024 Apr 30;12:RP91927. doi: 10.7554/eLife.91927 (PMC11060718; doi:10.7554/eLife.91927)
Supplement: Figure 3—source data 3. [file elife-91927-fig3-data3.zip › Figure 3 source data3 part 1.zip!/Figure 3-Source data 3-part1/1175-9-CRISPResso_Report_rtnd62ko.zip!/CRISPResso_on_1175_11/1c.Alignment_barplot.pdf]

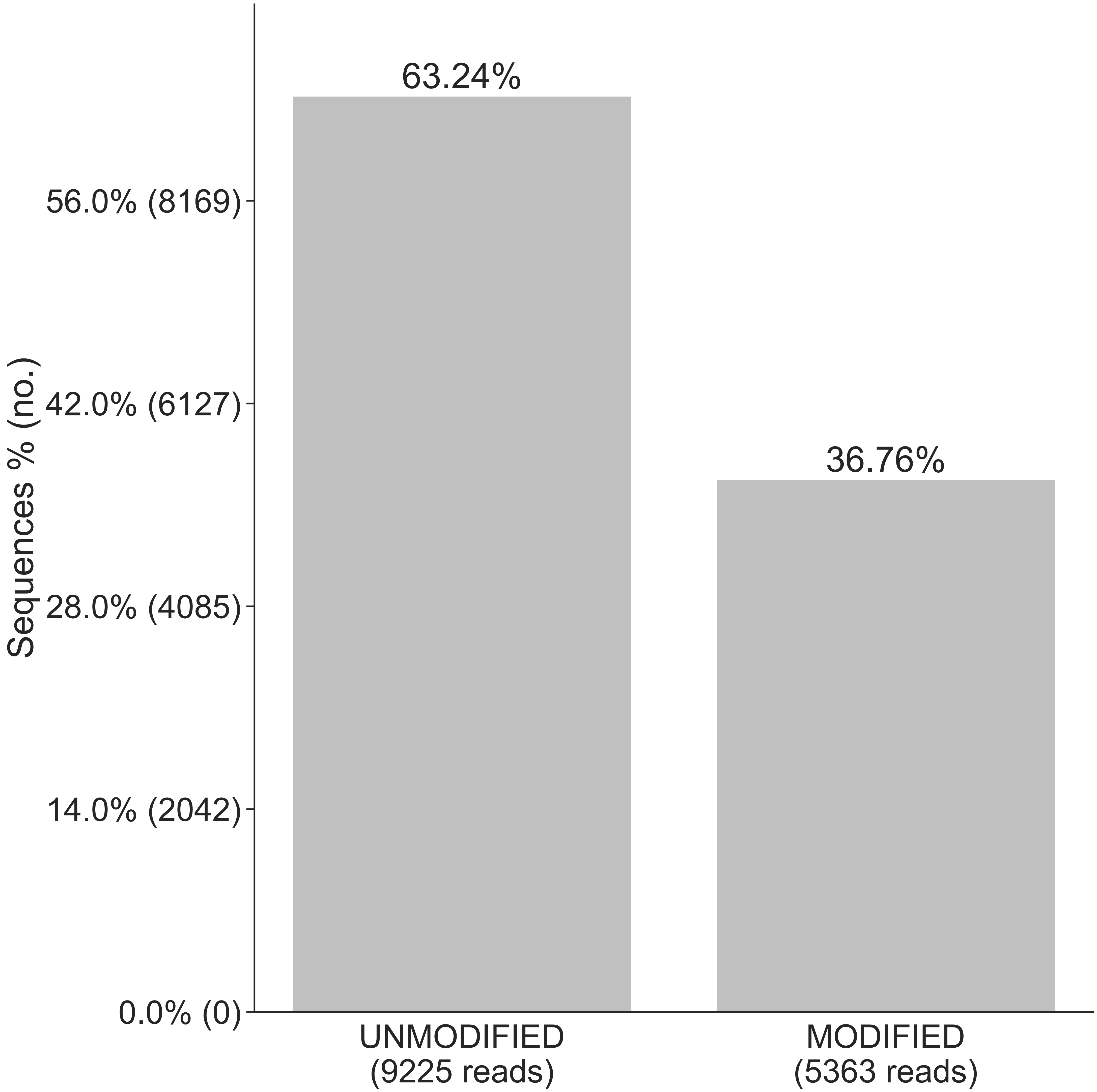
63.24%
36.76%
Sequences % (no.)
56.0% (8169)
42.0% (6127)
28.0% (4085)
14.0% (2042)
0.0% (0)
UNMODIFIED
(9225 reads)
MODIFIED
(5363 reads)
